# Evaluating Techniques for Vertical Ridge Augmentation via Comparative Study of Clinical Outcomes: A Systematic Review

**DOI:** 10.3390/jcm14248639

**Published:** 2025-12-05

**Authors:** Ioannis Frantzopoulos, Mihaela Băciuț, Oana Almășan, Avram Manea

**Affiliations:** 1 Faculty of Dentistry, Iuliu Hațieganu University of Medicine and Pharmacy, 400337 Cluj-Napoca, Romania; 2 Department of Maxillofacial Surgery and Implantology, Iuliu Hațieganu University of Medicine and Pharmacy, 400029 Cluj-Napoca, Romania; 3 Department of Prosthetic Dentistry and Dental Materials, Iuliu Hațieganu University of Medicine and Pharmacy, 400006 Cluj-Napoca, Romania

**Keywords:** alveolar ridge augmentation, bone regeneration, shell (Khoury) technique, cortical bone plate, titanium-reinforced d-PTFE membrane, implant survival, patient-reported outcomes

## Abstract

**Background/Objectives:** Vertical ridge augmentation (VRA) is often necessary in severe bone atrophy, yet the most predictable approach remains unclear. This systematic review compared Guided Bone Regeneration (GBR) and the Shell Technique (ST) for vertical bone gain (VBG), bone quality, complications, patient-reported outcomes (PROMs), and implant survival. **Methods:** Following PRISMA 2020 and PROSPERO registration (CRD420251128502), PubMed and Scopus databases were searched. Adults requiring VRA before implants were included. Interventions were GBR using titanium-reinforced dense PTFE (Polytetrafluoroethylene) or collagen membranes and ST using autogenous or allogeneic cortical plates. **Results:** Both techniques achieved clinically meaningful vertical augmentation. Median VBG was 4.24 mm for GBR (range 2.20–8.78 mm) and 5.16 mm for ST (range 3.10–7.60 mm) at re-entry (typically 4–9 months). Long-term series showed maintained gains for ST up to 10 years and multi-year stability after GBR in selected cohorts. Major early-healing complications were uncommon with both methods. Minor soft-tissue events varied; several GBR cohorts reported more exposures/dehiscence and occasional infections. Implant survival was uniformly high; validated PROMs were seldom reported. **Conclusions:** GBR and ST both enable vertical reconstruction sufficient for implant placement. ST tended toward higher median VBG but requires greater technical expertise and, when autogenous, adds donor-site morbidity; allogeneic shells reduce harvesting needs. GBR remains a versatile, donor-site-sparing alternative. Standardized outcome (including validated PROMs) reporting and head-to-head randomized trials are needed to refine case selection and confirm comparative effectiveness.

## 1. Introduction

Restoring vertical bone height is often essential for long-lasting, complication-resistant implant therapy [1]. Vertical ridge augmentation (VRA) is technically demanding, yet frequently required in patients with advanced atrophy resulting from periodontitis, trauma, or long-standing edentulism [1,2]. Because VRA procedures are highly technique sensitive, with a steep learning curve and non-trivial risk of healing complications, many clinicians favor graft-less solutions in selected cases, such as short implants, tilted or wide implants, zygomatic implants, or patient-specific subperiosteal implants [3,4,5,6,7,8]. When true vertical reconstruction is indicated, established augmentation methods include guided bone regeneration (GBR), the Shell Technique, customized/3D-printed titanium meshes, and alveolar distraction osteogenesis (ADO) [2,9,10,11]. These options frame a practical question: for durable, function-oriented rehabilitation, which strategy yields the most predictable vertical bone height with acceptable morbidity [12]?

Guided bone regeneration (GBR) uses barrier membranes to protect a grafted space and allow bone formation; modern protocols emphasize rigid space maintenance (e.g., titanium-reinforced PTFE membranes or titanium meshes) to attain clinically meaningful vertical gains [2,9,12]. At the same time, GBR is soft-tissue sensitive; early membrane or mesh exposure and dehiscence are the most frequent complications and are consistently associated with reduced vertical gain and compromised outcomes [13,14]. Contemporary reviews and meta-analyses corroborate both sides of this picture—predictable vertical gains with meshes and titanium-reinforced barriers, but non-negligible exposure rates that materially affect results [9,12,13,14].

The Shell Technique (Khoury concept) rebuilds thin cortical “walls” to create a rigid biologic container for particulate grafts, coupling space maintenance with favorable revascularization kinetics [10]. Autogenous shells are well documented; more recently, allogeneic cortical plates have been introduced to reduce donor-site morbidity. Early comparative data suggest similar short-term complication rates and shorter surgical time for allogeneic shells versus autogenous plates, with promising stability of the reconstructed ridge, although high-quality, long-term controlled evidence is still evolving [15,16].

Here, the literature is openly divided. Some authors, citing broad applicability and an extensive evidence base, treat GBR as the most versatile reference strategy for VRA [2,12], whereas others argue that the Shell Technique better preserves graft volume and reduces late resorption when soft-tissue handling is optimized, and that allogeneic shells can deliver comparable outcomes with less morbidity [10,15,16]. Adding to the disagreement, reports differ in defect morphology, barrier/graft choices, timing, and follow-up. Moreover, complications remain a major determinant of success across techniques, and patient-reported outcomes (PROMs), pain, swelling, and quality of life are still inconsistently reported, limiting truly patient-centered comparisons [17,18,19].

Despite decades of use, no clear consensus has emerged in favor of a single technique. This systematic review aims to address this gap by comparing GBR and the Shell Technique in vertical ridge augmentation, with a focus on vertical bone gain, bone density, complications, and patient satisfaction. The intention is to clarify where these methods overlap, where they diverge, and how they might best be integrated into a predictable, durable implantology workflow.

## 2. Materials and Methods

This systematic review was prepared according to the Preferred Reporting Items for Systematic Reviews and Meta-Analyses (PRISMA) 2020 guidelines (Supplementary Materials) [20]. The protocol was prospectively registered at PROSPERO (CRD420251128502). The authors acknowledge the use of ChatGPT (OpenAI, San Francisco, CA, USA) for language correction and editorial assistance during manuscript preparation. The authors reviewed and revised all AI-assisted content and accept full responsibility for the content of this publication. The research question, formulated according to the PICO framework, was as follows: In adult patients requiring implant therapy with vertical alveolar ridge deficiencies (P), how do Guided Bone Regeneration (GBR) procedures using dPTFE titanium-reinforced or Collagen membranes (I) compare with the Shell Technique (Khoury) using autogenous or allogeneic cortical plates (C) in terms of vertical bone gain, bone density/quality, complications, patient-reported outcomes, and implant survival (O)?

### 2.1. Eligibility Criteria

#### 2.1.1. Types of Studies

Eligible studies included human randomized controlled trials (RCTs), prospective and retrospective cohort studies, case–control studies, and other comparative clinical studies. Systematic reviews and meta-analyses were consulted for background only. Case reports, case series with fewer than 10 patients, animal studies, in vitro studies, conference abstracts, editorials, and narrative reviews were excluded.

#### 2.1.2. Population

We included adult, systemically healthy, non-smoking patients presenting with vertical alveolar ridge deficiencies of the maxilla or mandible requiring augmentation prior to implant placement. Studies focusing on augmentation for periodontal regeneration without implants, or in syndromic/cleft cases, were excluded.

#### 2.1.3. Intervention and Comparator

The GBR group comprised procedures employing dense PTFE (dPTFE), titanium-reinforced membranes, combined with particulate bone grafts (autogenous, allogeneic, xenogeneic, synthetic, or mixtures). Studies involving customized/3D-printed or CAD/CAM titanium meshes were excluded.

The Shell Technique group included procedures based on the Khoury concept, using thin autogenous cortical bone shells (intraoral donor sites) or allogeneic plates, filled with particulate grafts. Studies reporting conventional block grafts not consistent with shell principles were excluded.

#### 2.1.4. Outcomes Comprised

Studies reporting at least two of the following outcomes were eligible:

○ Primary outcome: vertical bone gain (mm).
○ Secondary outcomes: bone density/quality (histology, radiology, or clinical evaluation), complications (membrane/shell exposure, infection, graft loss, donor-site morbidity), patient-reported outcomes (pain, swelling, satisfaction), and implant survival (≥6 months post-loading).

#### 2.1.5. Time Frame and Language

Only full-text studies published in English between 2015 and 2025 were included. Minimum follow-up was ≥6 months post-augmentation, and ≥6 months after loading for implant survival outcomes.

### 2.2. Information Sources and Search Strategy

An electronic search was conducted in the following two databases, PubMed and Scopus, from 19 August 2025 to 15 September 2025. These two databases were selected a priori because they index the majority of peer-reviewed biomedical and dental journals and provide broad, complementary coverage of clinical trials and observational studies relevant to implant dentistry. No additional databases, trial registries, or gray literature sources were consulted. We acknowledge that restricting the search to two databases, English-language publications, and full-text articles may have led to the omission of relevant studies and represents a methodological limitation of this review:

○ PubMed: (Guided Bone Regeneration OR GBR) OR (Shell Technique OR Bone Blocks OR Bone Onlays) AND (Vertical Ridge Augmentation)
○ Scopus: TITLE-ABS-KEY (((guided AND bone AND regeneration OR gbr OR guided AND tissue AND regeneration) OR (shell AND technique OR bone AND blocks OR bone AND onlays OR bone AND transplantation) AND (vertical AND ridge AND augmentation OR alveolar AND ridge AND augmentation)))
  Filters: English language, publication years 2015–2025.

### 2.3. Study Selection and Data Collection

Two independent reviewers (I.F., A.M.) screened titles and abstracts against predefined inclusion/exclusion criteria. Potentially eligible studies were then assessed in full text. Disagreements were resolved by discussion or, if necessary, consultation with a third reviewer (MB). The screening process was managed in Zotero reference manager (version 7.0.29; Corporation for Digital Scholarship, Vienna, VA, USA), for de-duplication and Microsoft Excel (version 16.103.2 (25112216); Microsoft Corporation, Redmond, WA, USA) for tracking. During the preparation of this manuscript, the authors used ChatGPT Models GPT-4o and GPT-5o (OpenAI, San Francisco, CA, USA) for language correction, text polishing, and assistance with restructuring paragraphs for clarity. The tool was not used for data extraction, risk-of-bias assessment, or drawing scientific conclusions. After using this tool, the authors carefully reviewed and edited the content as needed and take full responsibility for the final version of the manuscript.

Data extraction was performed independently by three reviewers using a standardized Excel template (I.F., A.M., O.A.). Extracted variables included: study identifiers, design, country, sample size and demographics, defect characteristics, surgical details (membrane/shell, graft type, fixation, donor site, healing time), implant timing (simultaneous/staged), outcomes (bone gain, density/quality, complications, PROMs, implant survival), and follow-up durations.

### 2.4. Data Items

Operational definitions followed those of the original studies. “Vertical bone gain” was defined as the increase in alveolar ridge height (mm) measured clinically at re-entry and/or radiographically. Complications included any barrier/shell exposure, dehiscence, infection, graft failure, and donor-site morbidity. For the purposes of synthesis, all augmentation-healing complications were additionally reclassified into “major” and “minor” events at the patient level. Major complications were defined as events that compromised the augmentation or implant or required removal/re-augmentation or led to persistent morbidity beyond the expected postoperative course (e.g., partial or total graft loss, unplanned explantation, or lasting neurosensory deficit). Minor complications were defined as events that could be managed conservatively with full graft retention (e.g., small incision-line dehiscence, early membrane or lamina/screw exposures, localized soft-tissue inflammation) and did not materially affect graft success. PROMs included validated scales (e.g., VAS, OHIP) or qualitative patient reports when available.

### 2.5. Quality Assessment

In view of the clinical and methodological heterogeneity of the included studies, no single formal risk-of-bias tool was applied across all designs. Instead, risk of bias and overall methodological quality were assessed narratively. For each study, we considered domains broadly corresponding to standard risk-of-bias frameworks, including the clarity of inclusion criteria and defect description (selection bias), the adequacy of sample size and the presence or absence of appropriate comparators (where applicable), the handling of potential confounders, the transparency and reproducibility of outcome measurements (including imaging modality and landmark definition for vertical bone gain), the completeness of follow-up and reporting of withdrawals or losses to follow-up (attrition bias), and the consistency between reported outcomes and those described in the Section 2 (selective reporting). Particular attention was paid to study design (randomized controlled trial versus non-randomized cohort or case series), unit of analysis (patient, site, or implant), and the duration of follow-up for each outcome. Overall, most of the available evidence comes from non-randomized, single-arm, or observational cohorts with relatively small sample sizes and heterogeneous imaging and measurement protocols. Consequently, the collective risk of bias of the underlying literature is judged to be at least moderate, and in several domains potentially serious, which limits the strength of any comparative inferences. This limitation primarily reflects the current evidence base rather than the conduct of the present review.

### 2.6. Synthesis Methods

Given the clinical and methodological heterogeneity across included studies, quantitative pooling was not feasible. Data were synthesized using a narrative approach, supported by tabular presentation of study characteristics and outcomes. Comparative findings were discussed across five domains: vertical bone gain, bone density/quality, complications, PROMs, and implant survival. Exploratory subgroup contrasts (autogenous vs. allogeneic shells, simultaneous vs. staged implants, maxilla vs. mandible, short- vs. long-term follow-up) were summarized where reported. Vertical bone gain values were extracted as reported in each study, irrespective of the specific landmark used (for example, crest-to-graft height, effective bone height from the mandibular canal, or implant shoulder to first bone contact) and imaging modality (clinical re-entry probing/calipers, CBCT linear or superimposition methods, or panoramic radiographs with magnification correction). No attempt was made to mathematically standardize these different measurement protocols; instead, VBG was synthesized descriptively at the arm level, and methodological differences and outliers were highlighted qualitatively in the Results and Discussion when interpreting between-study heterogeneity. Consequently, the VBG values reported in this review should be interpreted as approximate contrasts across heterogeneous methodologies rather than as precise quantitative effect sizes when comparing ST and GBR.

## 3. Results

### 3.1. Data Collection

**Figure 1:**
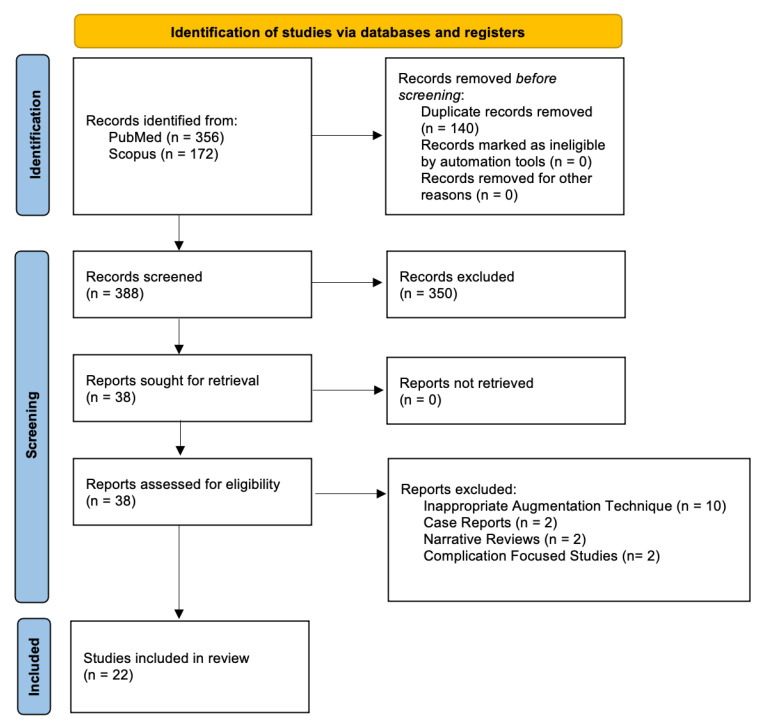
PRISMA flowchart.

A total of 528 records were identified across databases (PubMed 356, Scopus 172). After removing 140 duplicates (automation/other removals 0), 388 titles/abstracts were screened. In total, 350 records were excluded at this stage (not related to the topic or failing the eligibility criteria, background articles, reviews, case reports, or animal studies). We sought 38 full-text reports; 0 could not be retrieved, leaving 38 articles for full-text assessment. Following eligibility review, 10 were excluded for inappropriate technique, 2 were case reports, 2 were narrative reviews, and 2 only reported complications. Twenty-two studies were therefore included in the qualitative synthesis. The PRISMA flow is depicted in Figure 1.

### 3.2. Description of the Studies

This comparative review focuses on vertical ridge augmentation outcomes for the ST versus GBR across the 22 included studies [21,22,23,24,25,26,27,28,29,30,31,32,33,34,35,36,37,38,39,40,41,42]. Data were extracted using a standardized form capturing: (1) authors/year; (2) country/setting; (3) study design; (4) sample size and unit of analysis (patient/site/implant); (5) technique details (autogenous or allogeneic cortical plates and particulate blends for Shell; non-resorbable Ti-reinforced PTFE variants or resorbable collagen membranes with particulate grafts for GBR); (6) reference landmarks and measurement modality (clinical re-entry probing/calipers, CBCT linear or superimposition methods, occasionally panoramic with magnification correction); (7) vertical bone gain (VBG) at the study-specified timepoint; (8) complications (exposure/dehiscence, infection, graft failure/partial loss, donor-site morbidity, and other adverse events); (9) bone density surrogates (histology/histomorphometry when available); (10) patient-reported outcomes (PROMs); and (11) implant survival and, when provided, success criteria.

Most studies reported VBG at re-entry between 4 and 9 months, or early post-loading, with several cohorts providing longer-term remodeling/stability up to 10 years [21,22,23,24,25,26,27,28,29,30,31,32,33,34,35,36,37,38,39,40,41,42]. Because designs, timepoints, landmarks, units of analysis, and denominators (patient vs. site) varied substantially—and validated PROM instruments and quantitative density metrics were infrequently used—meta-analysis was not performed. Instead, we prespecified a comparative, arm-level synthesis: mixed-technique studies contributed separate arms to ST and GBR; complication rates were treated as patient-level when reported; and, per protocol for cross-study consistency, dehiscence was treated as synonymous with exposure. The results that follow are therefore presented outcome-by-outcome (VBG, complications, bone density, PROMs, implant survival), with medians/ranges emphasized and interpretive provided, acknowledging context the overall heterogeneity (Table 1) [21,22,23,24,25,26,27,28,29,30,31,32,33,34,35,36,37,38,39,40,41,42].

### 3.3. Study Characteristics

The selected studies on vertical ridge augmentation (VRA) primarily compared Guided Bone Regeneration (GBR) and the Shell Technique (ST) for achieving vertical bone gain, with follow-up periods ranging from 6 months to 10 years. The studies were conducted in multiple countries, including Germany, Italy, Turkey, and Egypt, and included both randomized clinical trials and prospective cohort studies.

The sample sizes varied from 10 to 372 patients, with vertical bone gain (VBG) ranging from 2.2 mm to 8.78 mm for GBR and 3.1 mm to 7.6 mm for ST. Most studies reported high implant survival rates, typically 100%, though a few studies did not report this data. Complications, particularly soft tissue issues like graft exposure or dehiscence, were reported in some studies, with minor complications occurring in up to 31.8% of GBR cases and up to 14.3% of ST cases. Major complications were rare, with few studies reporting graft failures or infections.

The follow-up periods varied, with many studies reporting results at 1 year post-loading, while others provided long-term data up to 10 years. 

#### 3.3.1. Results of Syntheses

##### Vertical Bone Gain

We synthesized arm-level vertical bone gain (VBG) from each study arm and summarized results by technique (no meta-analysis; heterogeneous designs/timepoints). Values are reported at the study-specified evaluation (most commonly re-entry at 4–9 months or early post-loading), rounded to 2 decimals.

##### Overall Comparison

Both techniques, the Guided Bone Regeneration (GBR) and the Shell Technique (ST), achieved clinically meaningful vertical augmentation across the included arms.

For GBR, a total of 9 arms were analyzed, with a median vertical bone gain (VBG) of 4.24 mm (ranging from 2.20 to 8.78 mm). The representative arm means varied, with the lowest being 2.20 mm in a split-mouth GBR arm using a resorbable collagen membrane [40], and the highest being 8.78 mm in an arm using Ti-reinforced d-PTFE with CBCT imaging [30]. Other representative means included 2.80 mm with ePTFE and a custom stent [31], 3.56 mm with Ti-reinforced PTFE [38], 4.20 mm with Ti-reinforced d-PTFE at clinical re-entry [24], 4.24 mm with Ti-reinforced d-PTFE using CBCT [36], 4.80 mm with MP-ePTFE via CBCT superimposition [35], 5.07 mm with d-PTFE using CBCT [33], and 5.78 mm with unfixed collagen using long-term CBCT [37].

For the Shell Technique (ST), a total of 8 arms were evaluated, with a median VBG of 5.16 mm (ranging from 3.10 to 7.60 mm). The representative arm means were 3.10 mm for autogenous shells with simultaneous implants using panoramic and caliper measurements [21], 4.20 mm for Khoury shells using CBCT [38], 4.70 mm for allogeneic shells with CBCT [26], 5.12 mm for bilaminar grafts in the anterior maxilla with clinical caliper measurements [23], 5.20 mm for the tunnel “box” technique with EBH measurements [41], 7.30 mm for posterior maxilla SBB + tunnel using clinical probing [22], 7.40 mm for posterior mandible SBB + tunnel with clinical probing [5], and 7.60 mm for split-block autogenous grafts with CBCT registration [32].

The VBG measuring techniques varied and included clinical re-entry probing/calipers [22,24,25,31], CBCT linear measures or superimposition [26,30,32,33,35,36,37,38], and panoramic radiographs with magnification correction [21,22,41].

Landmark definitions also differed (e.g., implant should as the first bone contact [24], vertical lines at buccal/mid/lingual [35], crest like the mandibular canal “effective bone height” (EBH) [41]). These differences introduce measurement heterogeneity, limiting direct between-study comparability. In particular, the highest GBR gain (8.78 mm in Scavia 2021) was obtained with CBCT superimposition over a defined region of interest [30], which is not directly comparable to the predominantly caliper- or probing-based measurements used in several Shell cohorts [22,25,32]; this single-arm outlier should therefore be interpreted as a method- and defect-specific result rather than as evidence that GBR generally achieves greater vertical augmentation than Shell.

##### Timing of Assessment

Most arms reported VBG at re-entry between 4 and 9 months (e.g., 4–6 months [23,28,32], 6 months [30,31,35,38], 6–9 months [33]) or 12 months post-loading [21,24,40,42]. Several cohorts also documented stability/remodeling after implant placement: for GBR, examples include 1-year remodeling of ~0.59 mm after a mean gain of 8.78 mm [30] and stable crestal levels up to 7 years with long-term VBG ~5.78 mm [37]. For Shell/SBB, long-term series showed small cumulative vertical resorption yet high retained gains at 10 years [22,25]. However, it should be noted that the multi-year GBR data derive from a limited number of cohorts with heterogeneous designs and follow-up periods (roughly 1–7 years), whereas the decade-long stability signal for Shell is based on larger, dedicated series in both posterior maxilla and mandible [22,25,30,37].

##### Direction of Effect

**Figure 2:**
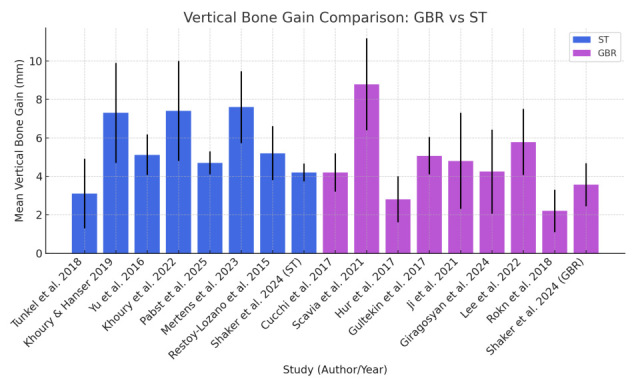
Comparison of mean vertical bone gain (VBG) between Guided Bone Regeneration (GBR) and the Shell Technique (ST) across included studies [21,22,23,24,25,26,30,31,32,33,33,35,36,37,38,40,41].

On arm-level medians, Shell showed a higher central tendency (5.16 mm) than GBR (4.24 mm). Ranges overlapped broadly (Shell 3.10–7.60 mm; GBR 2.20–8.78 mm), and the largest single gains were reported in both groups (e.g., GBR up to 8.78 mm [30], Shell 7.60 mm [32]) (Figure 2).

In Hur 2017 [31] (GBR), premature exposures/infection were associated with greater vertical loss during submerged healing, underscoring the impact of soft-tissue events on maintained VBG.

In the long-term Shell series, early gains around 7–8 mm were largely maintained (e.g., stable vertical gain, about 6.8–6.7 mm at 10 years) [22,25].

Restoy-Lozano 2015 [41] reported EBH (crest to mandibular canal) rather than a pure crest to graft landmark; we retained it because the study explicitly used EBH to quantify effective vertical augmentation within a standardized tunnel technique. Readers should note that EBH is a proxy and may not match the linear crest-to-graft convention used elsewhere.

#### 3.3.2. Complications

To avoid mixing severities, we re-extracted complications at the patient level using consistent definitions: major, events that compromised the augmentation/implant or required removal/persistent morbidity; minor, events managed conservatively with full graft retention. Given heterogeneous reporting, results are presented descriptively without pooling and aligned to each arm’s reported major/minor rates in their table.

##### Shell Technique Complications

Major complications were uncommon across shell or autogenous block reconstructions: 0% in Tunkel 2018 [21], Yu 2016 [23], Khoury & Hanser 2019 [22], Pabst 2025 (allogeneic shell arm) [26], Mounir 2021 [29], Mertens 2023 (autogenous blocks) [32], and Shaker 2024 (Khoury bone shell arm) [38]; 0.9% in Khoury 2022 (one graft failure during healing) [25]; 1.1% in Kämmerer 2022 (cases preventing implant placement) [28]; and 4.7% in Restoy-Lozano 2015 (two early graft losses due to lamina mobility) [41].

Minor events, mainly small incision-line dehiscence or early site-level screw/exposure, ranged from 0% to 14.3%, with representative arm-level rates of 4.9% (Khoury & Hanser 2019) [22], 4.8% (Yu 2016) [23], 1.7% (Khoury 2022) [25], 10.0% (Pabst 2025) [26], 7.0% (Kämmerer 2022) [28], 14.3% (Mounir 2021) [29], 12.7% (Mertens 2023) [32], and 12.5% (Shaker 2024 shell arm) [38]. Although site-level screw or lamina exposures were frequent in some series (approximately 20% of sites in 2019 and 24% in 2022) [22,25], these events were usually managed by local hygiene and soft-tissue adjustment without graft removal and therefore contributed to the minor-complication category in our patient-level synthesis.

Guided Bone Regeneration (GBR) Complications

Where studies reported analyzable *patient-level* data with early-healing severity, major complications—events that led to partial or total graft/implant loss or required re-intervention—ranged from 0% to 10%. A 10% major rate was observed in the RCT by Cucchi 2017, where early failures were attributed to graft or implant loss during healing [24]. In contrast, no major augmentation-healing events were reported in Scavia 2021 [30], Hur 2017 [31], Gultekin 2017 (vertical subgroup) [33], Lee 2022 [37], Shaker 2024 (Ti-reinforced PTFE arm) [38], or Rokn 2018 [40]. Cucchi 2020, reporting the 3-year follow-up of the same RCT cohort, reiterated the original early-healing classification (10% major, 5% minor in the d-PTFE arm) without additional early-healing events [42].

Minor *patient-level* complications—predominantly small incision-line dehiscence or early membrane/mesh exposure, sometimes with localized soft-tissue inflammation but managed conservatively with full graft retention—varied widely across GBR arms: 5.0% in Cucchi 2017 [24], 10.7% in Scavia 2021 [30], 25.0% in Hur 2017 [31], 4.8% in Gultekin 2017 [33], 31.8% in Lee 2022 [37], 0% in the Shaker 2024 GBR arm [38], and 50.0% in the augmented sides of the split-mouth design by Rokn 2018 [40]. Several of these GBR cohorts also described exposures at the *site* level, which were more frequent than the corresponding patient-level rates because individual patients contributed multiple sites; where only site-level data were available, or severity could not be reliably stratified, studies were summarized separately and not included in the major/minor rate synthesis.

##### Non-Reporting/Not Classifiable for Early-Healing Severity

Several reports did not provide analyzable major/minor separation for augmentation-healing: Ji 2021 (site-level exposures reported but not patient-level severity rates) [35]; Giragosyan 2024 (Fontana classes shown graphically without counts) [36]; Cucchi 2023 (3-year follow-up cites earlier trial’s early-healing rates only) [34]; Cucchi 2019 (histology only) [39]; and Tatli 2025 (long-term peri-implant disease; no augmentation-healing events) [27]. These were not used in the major-rate narrative.

##### Timing and Patterns

**Figure 3:**
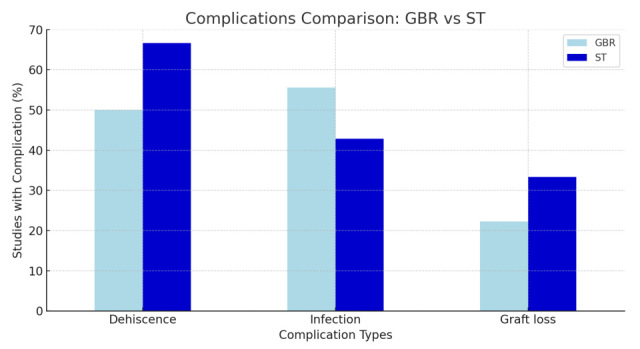
Complications Comparison: GBR vs. ST (observe the similarities).

Minor events clustered early (first 2–8 weeks) as small incision-line dehiscence or early barrier/screw exposures in both shell and GBR cohorts [22,23,24,25,30,31,37,38,41,42]. Some late exposures appeared around ~4 months in GBR cohorts (e.g., Scavia 2021; Gultekin 2017) [30,33]. Overall, major augmentation-healing complications were uncommon in both technique groups; observed differences were driven primarily by the frequency of minor, early soft-tissue events rather than by graft failures (Figure 3).

#### 3.3.3. Bone Density

Across arms, quantitative radiographic density was not reported; most studies focused on linear vertical gain and stability/resorption rather than density per se [21,22,23,24,25,26,27,30,31,32,33,34,36,37,38,40,41,42]. A minority provided histology/histomorphometry at re-entry/implant placement.

##### ST

Kämmerer et al., 2022 [28] conducted a qualitative histological evaluation using H&E and Masson–Goldner staining for allogeneic shell grafts. Their findings indicated advanced osteogenesis with lamellar bone formation and vascularized trabeculae around the embedded graft particles, with minimal inflammation and necrosis. However, no quantitative percentage of bone area was reported. Similarly, Mounir et al., 2021 [29] assessed autogenous shells and reported a significant difference in bone area percentage between different donor sites at 6 months post-augmentation. The chin grafts showed a bone area percentage of 52.53 ± 1.68%, while the retromolar grafts showed 47.97 ± 1.83%. Both grafts indicated well-consolidated bone, as confirmed by histology (H&E) and image analysis.

##### GBR

Ji et al., 2021 [35] evaluated the effectiveness of d-PTFE membranes and found that at 6 months post-augmentation, the regenerated bone comprised 34.91 ± 11.61% new bone, with 7.16 ± 2.74% residual graft material and 57.93 ± 11.09% soft tissue. This histological analysis was performed using trephine cores and H&E staining.

Lee et al., 2022 [37] studied the use of unfixed collagen membranes, providing qualitative histology on a small subset of samples. The analysis described new bone formation around residual biomaterial with marrow-like tissue, but did not include quantitative percentages. Finally, Cucchi et al. (2019) [39] employed Ti-reinforced d-PTFE membranes and assessed histomorphometry at 9 months post-augmentation. They found that the regenerated area (ROI-1) contained 39.7 ± 11.4% new bone, 8.6 ± 6.6% residual graft material, and 52.1 ± 13.7% soft tissue.

#### 3.3.4. Patient-Reported Outcomes (PROMs)

PROMs were infrequently and inconsistently captured. Most arms relied on narrative postoperative descriptions (pain, swelling, discomfort, acceptance) without validated instruments (e.g., VAS, OHIP); this was common in both Shell and GBR arms [21,23,24,28,29,31,32,33,34,35,36,37,38,40,41,42].

Notable exceptions include two large Shell cohorts using a structured analgesic-consumption classification at 2 weeks (suture removal): in 2019 (posterior maxilla, SBB + tunnel), little pain 40.1%, moderate 57.8%, heavy 2.1% [22]; in 2022 (posterior mandible, SBB + tunnel), little 54.7%, moderate 43.6%, heavy 1.7% [25]. An allogeneic Shell series reported no donor-site morbidity and one small dehiscence managed conservatively [26]; autogenous Shell studies occasionally noted transient donor-site events (e.g., bleeding, paresthesia) that resolved [29].

Among GBR arms, reporting was largely clinician-based (e.g., “discomfort in complication cases,” “reduced healing time,” “delayed healing/sloughing”) rather than patient-scale measures [24,30,31,33,34,35,36,37,38,39,41,42]. One long-term GBR cohort used Pink Esthetic Score (PES) (8.16 ± 1.35 at ~6 years), which is clinician-reported rather than a true PROM [27]. Overall, the PROM dataset was sparse, mostly narrative, and relied on non-standardized instruments, so the absence of consistent patient-centered metrics substantially limits the clinical significance of any comparative conclusions that can be drawn between ST and GBR.

#### 3.3.5. Implant Survival

For the ST, the arm-level survival rates reported across various studies are as follows: Tunkel 2018 [21] reported a 100.0% survival rate at 12 months, Khoury & Hanser 2019 [22] reported 98.9% at 120 months, and Yu 2016 [23] observed a 100.0% survival rate at approximately 72 months. In 2022, Khoury & Hanser [25] again reported a survival rate of 98.2% at 120 months. Kämmerer 2022 [28] observed a survival rate of 99.4% across a range of 12 to 144 months, with a mean of approximately 42 months. Mertens 2023 [32] reported a 100.0% survival rate across a follow-up period of 12 to 84 months, with a mean of approximately 29 months. Restoy-Lozano 2015 [41] also found a 100.0% survival rate at 18 to 54 months, with a mean of about 33 months. Shaker 2024 [38] reported a 100.0% survival rate at 3 months for the Shell arm. It should be noted that Pabst 2025 [26] and Mounir 2021 [29] did not report survival data for their respective studies.

For GBR, the arm-level survival rates reported are as follows: Cucchi 2017 [24] found a 100.0% survival rate at 12 months, Gultekin 2017 [33] observed a 100.0% survival rate at 18 to 39 months, with a mean of approximately 31 months, and Cucchi 2023 [34] also reported 100.0% survival at 36 months. Ji 2021 [35] reported 100.0% survival at 12 months, while Lee 2022 [37] reported 100.0% survival at 12 to 84 months, with a mean of approximately 70 months, though one early loss was replaced. Shaker 2024 [38] reported a 100.0% survival rate at 3 months for the GBR arm. Rokn 2018 [40] also found a 100.0% survival rate at 12 months, while Tatli 2025 [27] observed 99.2% survival at an average follow-up of about 72 months. Cucchi 2020 [42] reported a 100.0% survival rate at 12 months. Survival data were not reported for Scavia 2021 [30], Hur 2017 [31], nor Giragosyan 2024 [36].

In terms of overall survival across the studies:

For the Shell Technique, 8 arms with survival data were analyzed, with a median survival rate of 100.0% (range 98.2–100.0%) and follow-up ranging from 3 to 120 months.

For Guided Bone Regeneration, 9 arms with survival data were analyzed, with a median survival rate of 100.0% (range 99.2–100.0%) and follow-up ranging from 12 to 120 months.

Taken together, these data show that, within the follow-up periods reported, including a limited number of cohorts with up to 10 years of observation, implants placed in conjunction with either ST or GBR can reach high survival levels. However, because implant survival is strongly influenced by multiple patient-, site-, and prosthetic-related factors and not solely by the augmentation technique, these values should be viewed primarily as general confirmation of the long-term feasibility of implant placement in vertically augmented ridges rather than as a direct comparison between the two approaches.

## 4. Discussion

### 4.1. Principal Findings and Clinical Positioning

This comparative review asked a pragmatic question for clinicians routinely treating severe vertical atrophy: between the ST and GBR, which approach is easier to perform, more predictable, associated with fewer complications, and yields the most favorable long-term outcomes. Synthesizing arm-level data from 22 included studies, we found that both techniques reliably achieve clinically meaningful vertical bone gain sufficient for implant placement, with Shell showing a higher median VBG (5.16 mm) than GBR (4.24 mm), albeit with broad overlap and measurement heterogeneity [21,22,23,24,25,26,28,29,30,31,32,33,35,36,37,38,40,41]. Complications clustered around exposure/dehiscence for both, with GBR arms more frequently reporting infection and autogenous Shell showing donor-site morbidity in some series [22,24,25,28,29,30,31,32,33,35,36,37,38,40,41]. Implant survival was uniformly high for both techniques across reported follow-ups [21,22,23,24,25,28,32,33,34,35,37,38,39,40,41,42]. Overall, our interpretation is moderately confident but remains descriptive given the preponderance of non-randomized evidence and methodological variability. From a risk-of-bias perspective, the predominance of observational designs, variable imaging methodologies, and incomplete reporting of patient- and defect-level confounders mean that the comparative findings in this review should be interpreted with appropriate caution rather than definitive evidence of superiority for either technique.

### 4.2. Vertical Bone Gain and Maintenance over Time

Across arms, both techniques delivered approximately 3–8 mm of vertical augmentation at the timepoints measured (typically re-entry at 4–9 months or early post-loading) [21,22,23,24,25,26,28,29,30,31,32,33,35,36,37,38,40,41]. The Shell Technique reached a higher median VBG in our arm-level synthesis, while GBR included single-arm outliers with very high linear gains (e.g., approximately 8.8 mm) [30]. Methodology matters; studies used clinical re-entry probing/calipers [22,24,25,31], CBCT linear or superimposition [26,30,32,33,35,36,37,38], and panoramic with magnification correction [21,22,41], with differing landmarks, and these differences limit direct comparability. For example, the approximately 8.8 mm GBR gain reported by Scavia 2021 [30] was calculated using CBCT superimposition and a defined volumetric region, whereas the largest Shell gains (around 7–8 mm) were mostly derived from clinical probing or caliper measurements at re-entry or from effective bone height (EBH) metrics [22,25,32,41]. Such differences in imaging modality, landmark choice, and defect morphology mean that absolute VBG values across techniques cannot be assumed to be directly comparable, and the observed central tendency favoring Shell in this review should be understood as a broad trend within heterogeneous methodologies rather than as a precise, statistically validated effect size.

Long-term stability is crucial. ST cohorts with extended follow-up in both posterior maxilla and mandible reported retained vertical gains at 10 years with modest cumulative resorption and broadly consistent behavior across series [22,25,32,41,43]. In contrast, long-term GBR data in this review are restricted to a smaller number of cohorts, typically with follow-up between 1 and 7 years, heterogeneous study designs, and variable reporting landmarks [30,34,37]. Within these constraints, the available GBR series suggest stable crestal levels and maintained augmentation over the medium term [30,34,37], but the time horizon is shorter and less consistent than for Shell. Clinically, this implies that Shell currently provides the most mature evidence base for decade-long ridge-level stability, whereas GBR appears promising for medium-term maintenance but requires additional, standardized long-term data before true equivalence in vertical stability can be assumed.

### 4.3. Complications: Patterns and Clinical Nuance

Using patient-level major/minor definitions (rather than lumping all events), major complications were uncommon in both Shell and GBR. Shell cohorts largely showed between 0% and 5% major events (highest in an early lamina series), while GBR ranged 0–10% (10% in one RCT), and ranges overlapped. Between-arm differences were driven primarily by minor, early soft-tissue events (small dehiscence/exposure) rather than graft failure [21,22,23,24,25,26,28,29,30,31,32,33,35,36,37,38,40,41,42].

#### 4.3.1. Exposure/Dehiscence

High-event GBR examples (e.g., site/patient-level soft-tissue complications or maintained exposures) contrasted with lower-event series: GBR examples include notable soft-tissue complications in Lee 2022 and frequent augmented-side events in a split-mouth design, while other GBR cohorts reported far fewer minor events; Shell examples included frequent site-level screw exposures with low patient-level failure (e.g., ~20–24% sites in long-term shell series) [22,25,30,33,35,37,40].

#### 4.3.2. Infection

Infection appeared more often in GBR reports (e.g., RCT and cohort data) than in Shell cohorts, where it was sporadic (isolated cases with or without subsequent failure) [22,24,25,30,31,35].

#### 4.3.3. Donor-Site Morbidity

Donor-site morbidity is unique to autogenous Shell/blocks and was generally transient (bleeding/paresthesia resolving within weeks); allogeneic Shell avoids donor harvesting and showed low complication profiles in the included cohorts [26,28,29,32].

Notably, several Shell series reporting minor complications still permitted implant placement at re-entry in most cases; outright early graft failure remained the exception (e.g., select lamina instability or rare infection-related loss) [28,41].

Minor early exposures/dehiscences were commonly amenable to conservative care (meticulous hygiene/chlorhexidine, selective trimming or barrier removal when indicated), while tension-free closure and careful soft-tissue handling remained central determinants of uneventful healing across both techniques [22,24,25,30,31,35,37,38,40,41,42,44,45] (Figure 3).

### 4.4. Technique Considerations: Autogenous vs. Allogeneic Shell; Ti-PTFE vs. Collagen GBR

Within Shell, allogeneic cortical plates offer a less invasive option (no second surgical field), reduced donor-site morbidity, and acceptable complication profiles in larger series [26,28]. Autogenous Shell remains a powerful option with long-term stability data [22,25,32,41], but entails harvesting-related morbidity [29,32].

Historically, vertical augmentation of the posterior mandible relied heavily on conventional block grafting, either as onlay autogenous blocks or interpositional/inlay blocks. A CBCT-based comparative study by Barone et al. evaluated interpositional equine cancellous blocks versus autogenous onlay blocks in atrophic posterior mandibles and reported vertical augmentation heights of around 6.0–7.4 mm at surgery, followed by volumetric losses of approximately 29–35% and vertical reductions of about 1.7–1.9 mm over a 4-month healing period. Clinical complications included wound dehiscence and, in one case, mandibular fracture, and the success of autogenous onlay blocks (82.4%) was lower than that of inlay grafts (93.8%), although the difference was not statistically significant. These findings illustrate both the biological potential and the limitations of traditional block grafting—substantial early remodeling and non-trivial morbidity—and help contextualize the evolution toward thinner cortical shell concepts that aim to reduce bulk and donor-site burden while maintaining vertical stability [46].

Within GBR, Ti-reinforced PTFE membranes dominate the vertical applications in our set [24,30,31,34,35,36,38,39,42], while resorbable collagen membranes were used in selected contexts (including unfixed collagen for vertical applications) with encouraging long-term ridge maintenance [37]. Techniques popularized for collagen-based GBR (e.g., “sausage”-style space maintenance) sit conceptually adjacent [2,47], but most vertical GBR in our dataset relied on rigid, Ti-reinforced barriers for predictable space creation.

Simultaneous vs. staged implants. Consistent with your stance, staged placement was the default in most vertical cases; although simultaneous placement appears in selected Shell series [21], vertical augmentation typically benefits from staging to de-risk complications and optimize soft-tissue management.

### 4.5. PROMs: A Critical Evidence Gap

Patient-reported outcomes were rarely captured with validated instruments in either technique, which substantially limits how confidently the comparative findings of this review can be translated into patient-centered decisions and reduces the clinical significance of many between-technique differences. Two large ST cohorts used analgesic-consumption classes at 2 weeks and showed predominantly little-to-moderate pain [22,25]; otherwise, PROMs reporting was narrative (discomfort, delayed healing, acceptance) across both Shell and GBR arms [21,23,24,28,29,31,32,33,34,35,36,37,38,40,41,42]. This pattern aligns with broader observations that PROMs in regenerative dentistry are often underused or heterogeneously recorded, despite evidence that patient-reported pain, function, and quality of life can diverge from clinician-assessed surrogate outcomes [48]. Future VRA trials should therefore make the use of validated PROM instruments (e.g., VAS-based pain scales, OHIP-derived oral health-related quality-of-life indices) mandatory and report them systematically alongside clinician-reported outcomes, so that differences in vertical gain and complication profiles can be directly linked to patients’ experience of treatment burden and benefit [48].

### 4.6. Implant Survival as a Feasibility Indicator

Reported implant survival was excellent in all included series, with median values of 100.0% and arm-level ranges of 98.2–100.0% for Shell and 99.2–100.0% for GBR across follow-up periods extending from a few months up to 10 years [21,22,23,24,25,28,32,33,34,35,37,38,39,40,41,42]. This near-uniform, ceiling effect indicates that, in appropriately selected cases, implants placed in conjunction with either augmentation strategy can achieve predictable osseointegration and long-term function. At the same time, such clustered survival rates are inherently non-discriminatory and should not be interpreted as evidence that one technique is superior to the other or as a direct reflection of the augmentation method itself. Because survival was usually reported as a binary outcome and “success” (e.g., marginal bone level thresholds, absence of complications) was not uniformly defined, these data primarily confirm overall clinical feasibility rather than provide a fine-grained performance signal.

Beyond the descriptive medians, it is important to recognize that implant survival is multifactorial: patient-related factors (e.g., smoking, systemic conditions), site-related variables (bone quality and quantity), and treatment variables (e.g., immediate vs. healed placement, implant geometry and surface, prosthetic loading protocols) all materially influence failure risk, so attributing differences in survival solely to the augmentation technique is inappropriate [43,49,50,51]. Available long-term series, such as 10-year Shell cohorts, remain relatively few but suggest that high survival can be maintained over extended follow-up when cases are carefully selected and executed [22].

Large clinical datasets further report comparable, and in some series slightly higher, survival in augmented versus native sites, reinforcing that the augmentation method per se is not the dominant driver of implant loss [51]. Moreover, high-level reviews conclude that no single augmentation technique can be identified as clearly superior in terms of survival outcomes, and alternatives such as short implants may achieve similar survival in selected indications [43,52]. In this context, ridge-level stability and complication patterns, particularly the maintenance of vertical gain over multi-year and decade-long follow-up, as discussed in Section 4.2, are likely more informative than implant survival alone when choosing between ST and GBR in daily practice.

### 4.7. Concept Note: Resorbable Collagen Membrane as a Soft-Tissue Stimulant

Within the vertical context of this review, we propose (but do not demonstrate) a possible “collagen-as soft-tissue-spacer” concept. After vertical augmentation with Ti-reinforced GBR, a resorbable collagen membrane could be positioned not only as a barrier but also as a deliberate spacer, aiming to maintain a protected vertical clot compartment that matures into thicker mucosa and/or increased keratinized tissue width without the need for a concomitant connective-tissue graft (CTG).

In our 22 included studies, however, no arm was specifically designed to test this indication, and none reported pre–post quantitative soft-tissue changes attributable solely to a membrane placed as a vertical soft-tissue spacer. Yu 2016 [23] used double collagen membranes over Shell grafts and noted that a simplified flap “can augment the keratinized tissue,” but no keratinized tissue or mucosal-thickness measurements were provided. Lee 2022 [37] reported long-term hard-tissue stability and dense connective tissue at GBR sites with unfixed collagen membranes, but again without quantitative soft-tissue outcomes. Where soft-tissue gains were measured, they were typically linked to planned CTG (e.g., in the randomized trial by Cucchi and colleagues) rather than to a membrane-only effect [34,42].

Biologically, the idea of a collagen membrane acting as a temporary soft-tissue spacer is plausible—by creating and protecting a blood clot, allowing granulation, and guiding mucosal maturation during membrane resorption—but on the basis of the current evidence, it remains a hypothesis only. It should therefore not be regarded as an established therapeutic strategy. Instead, it represents a pragmatic, low-morbidity concept that may warrant formal testing in future prospective studies, particularly in situations where additional soft-tissue volume would be beneficial for sealing, exposure resistance, and maintenance of peri-implant health, yet surgeons wish to avoid CTG at the primary augmentation stage [23,28,34,37,42].

Outside the vertical augmentation setting, additional support for the biological behavior of DBBM combined with collagen matrices comes from socket-preservation data. In a prospective study, Maiorana et al., 2017 [53] reported that post-extraction sockets grafted with demineralized bovine bone mineral and covered by a porcine-derived collagen matrix showed minimal dimensional changes over 6 months (mean vertical loss ≈ 0.46 mm and horizontal loss ≈ 1.21 mm), with all sites healing uneventfully to allow implant placement. Histologic and histomorphometry analyses demonstrated newly formed bone in close contact with residual DBBM particles and an overlying keratinized mucosa supported by dense, vascularized, collagen-rich connective tissue. These findings reinforce the concept that collagen matrices can stabilize the blood coagulum, guide soft-tissue maturation, and act as low-morbidity soft-tissue substitutes, even though their deliberate use as vertical “spacers” in VRA remains a hypothesis that still requires direct clinical testing [53].

### 4.8. Practical Guidance: Choosing Between Shell and GBR

When vertical demand is high (≈≥5–7 mm) and soft tissues allow tension-free management, Shell (autogenous or allogeneic) is a strong candidate, supported by long-term stability data [22,25,32,41]. In cases where avoiding donor-site morbidity is crucial, such as patients on anticoagulant therapy, those with medical complexities, or when patientpreference strongly favors minimally invasive approaches, an allogeneic Shell or GBR becomes highly attractive. The allogeneic Shell eliminates harvesting while maintaining good outcomes [26,28].

GBR remains versatile; Ti-reinforced PTFE offers predictable space maintenance for vertical defects, while collagen-based GBR (including unfixed approaches) may be considered in selected scenarios with careful soft-tissue handling [30,31,33,34,35,36,37,38,42].

The Shell Technique, especially when using autogenous grafts, demands a steeper learning curve, requiring expert handling to avoid complications and over-manipulation [2,22,25,32,41].

For vertical reconstructions, staged implant placement is generally preferable, while simultaneous placement is reserved for carefully selected cases, following a decision framework adapted from techniques like Local Ridge Augmentation (LRA) [21,54].

### 4.9. Future Directions

There are several key priorities for future research in the field of vertical ridge augmentation. One important area is the need for head-to-head randomized controlled trials (RCTs) comparing autogenous versus allogeneic shell grafts and shell versus titanium-reinforced Guided Bone Regeneration (GBR) techniques. These studies should have follow-up periods of at least 12 to 36 months to ensure the long-term effectiveness and predictability of the techniques. Another priority is the development of core outcome sets that include uniform vertical bone gain (VBG) landmarks, standardized cone-beam computed tomography (CBCT) protocols, consistent timepoints for follow-up, and standardized exposure definitions across studies. This would enhance the comparability and robustness of results [21,22,23,24,25,26,27,28,29,30,31,32,33,34,35,36,37,38,39,40,41,42].

Additionally, it is crucial to mandate the use of validated Patient-Reported Outcome Measures (PROMs) in these studies and to explicitly report donor-site morbidity outcomes to provide a complete picture of the patient experience and potential complications. Another promising area of investigation is the testing of the “soft-tissue spacer” concept using collagen membranes. Furthermore, research should explore subgroups based on clinical variables such as the posterior mandible versus maxilla and thin versus thick biotypes. This would help refine case selection and ensure that the most appropriate augmentation technique is used based on the individual patient’s characteristics. These priorities are reflected in several studies that have emphasized the need for more targeted research [21,22,23,24,25,26,27,28,29,30,31,32,33,34,35,36,37,38,39,40,41,42].

### 4.10. Clinical Implication

From a clinical perspective, shell grafts, whether autogenous or allogeneic, are generally advised when vertical bone gain of ≥5–7 mm is necessary, and when the soft tissues allow for tension-free closure. In cases where the clinician wishes to avoid donor-site morbidity, GBR or allogeneic shell grafts may be a suitable alternative, particularly if the clinician has limited experience with the Shell Technique. For clinicians who are not yet proficient in the Shell Technique, opting for GBR or allogeneic shell grafts may be justifiable to build expertise over time. The decision-making process should also consider the concerns raised in many non-randomized studies, along with the overall heterogeneity in the literature. Given these considerations, there is a pressing need for more head-to-head randomized trials that directly compare shell grafts with GBR techniques. Such studies will be essential for refining case selection and confirming which technique provides the most predictable and durable outcomes in specific clinical scenarios. These trials will help clarify the most effective approach for different patient needs and clinical conditions.

## 5. Conclusions

Both ST and GBR reliably achieve clinically meaningful vertical augmentation sufficient for implant placement in severe atrophy. In our arm-level synthesis, ST showed a higher median vertical bone gain than GBR, and major early-healing complications were uncommon with both approaches. However, because the evidence base is dominated by non-randomized designs, uses heterogeneous imaging modalities and landmarks for vertical measurements, and features variable follow-up durations, no augmentation technique can be conclusively identified as superior. The observed trends towards higher median VBG and more mature decade-long ridge-level stability data in ST studies should therefore be interpreted with appropriate caution rather than as definitive proof of superiority. Implant survival was uniformly high across both techniques and, being multifactorial and near-ceiling, is largely non-discriminatory for comparative purposes. ST offers predictable vertical gain with long-term maintenance but requires greater operator expertise and, when autogenous, entails donor-site morbidity; allogeneic shells mitigate harvesting while maintaining favorable outcomes. GBR remains a versatile, donor-site-sparing alternative, particularly when donor-site avoidance is prioritized or when defects are narrower, but in the available literature, it tends to show higher rates of soft-tissue exposures/dehiscence and occasional infections. Evidence for PROMs is sparse, mostly narrative, and rarely based on validated instruments, so no technique-level advantage can be claimed from the patient’s perspective, and the clinical significance of observed differences in hard-tissue and complication outcomes remains incompletely defined. Future studies should therefore prioritize standardized outcome definitions (including VBG landmarks and complication grading), incorporate validated PROMs alongside clinician-reported outcomes, and use well-designed head-to-head randomized trials to refine case selection and confirm the comparative effectiveness of Shell and GBR.

## Figures and Tables

**Table 1:** Characteristics of included studies.

Author (Year)	Country/Center	Study Design	Technique	*n* (Patients)	Mean Vertical Gain (mm)	Complication Rate (%) Major/Minor	Bone Density Reported	Implant Survival (%)	Follow-Up Period
Tunkel et al., 2018 [21]	Germany/Italy	Retrospective case series	ST	10	3.1	0/0	No	100	1 year (post loading)
Khoury & Hanser 2019 [22]	Germany	Prospective clinical study	ST	142	7.3	0/4.9	No	98.9	10 years
Yu et al., 2016 [23]	China	Prospective clinical study	ST	21	5.12	0/4.8	No	100	6 years
Cucchi et al., 2017 [24]	Italy	Randomized clinical trial	GBR	20	4.2	10/5	No	100	1 year (after loading)
Khoury et al., 2022 [25]	Germany	Prospective clinical cohort	ST	117	7.4	0.9/1.7	No	98.2	10 years
Pabst et al., 2025 [26]	Germany, Austria, Saudi Arabia	Retrospective multicenter comparative cohort	ST	10	4.7	0/10	No	NR	1 year
Tatli et al., 2025 [27]	Turkey	Retrospective comparative cohort	GBR	127	NR	NR/NR	No	99.21	6 years
Kämmerer et al., 2022 [28]	Germany, Austria	Retrospective multicenter case series	ST	372	NR	1.1/7	Yes	99.39	1–12 years
Mounir et al., 2021 [29]	Egypt	Randomized clinical trial	ST	14	NR	0/14.3	Yes	NR	6 months
Scavia et al., 2021 [30]	Italy	Prospective case series	GBR	28	8.78	0/10.7	No	NR	1 year
Hur et al., 2017 [31]	USA	Prospective case series	GBR	16	2.8	0/25	No	NR	NR
Mertens et al., 2023 [32]	Germany	Retrospective comparative cohort	ST	55	7.6	0/12.7	No	100	1–7 years
Gultekin et al., 2017 [33]	Turkey	Retrospective cohort	GBR	21	5.07	0/4.8	No	100	1.5–3 years
Cucchi et al., 2023 [34]	Italy	Randomized clinical trial	GBR	15	NR	NR/NR	No	100	3 years
Ji et al., 2021 [35]	Korea	Prospective case series	GBR	14	4.8	NR/NR	Yes	100	1 year
Giragosyan et al., 2024 [36]	Bulgaria	Randomized controlled trial	GBR	20	4.24	NR/NR	No	NR	6 months
Lee et al., 2022 [37]	Korea	Retrospective cohort	GBR	22	5.78	0/31.8	Yes	100	1–7 years
Shaker et al., 2024 [38]	Egypt	Randomized clinical trial	ST & GBR	16	4.2 (ST); 3.56 (GBR)	0.0/12.5 (ST) 0.0/0.0 (GBR)	No	100% (both arms)	6 months
Cucchi et al., 2019 [39]	Italy	Randomized clinical trial	GBR	20	NR	NR/NR	Yes	NR	1 year
Rokn et al., 2018 [40]	Iran	Randomized controlled trial	GBR	11	2.2	0/50	No	100	1 year
Restoy-Lozano et al., 2015 [41]	Spain	Prospective case series	ST	43	5.2	4.7/NR	No	100	1.5–4.5 years
Cucchi et al., 2020 [42]	Italy	Randomized controlled trial	GBR	20	NR	10/5	No	100	1 year (post loading)

Abbreviations: GBR, Guided Bone Regeneration; ST, Shell Technique; NR, not reported. Complication rate reported as major/minor (%). Major = events compromising augmentation/implant or requiring removal; Minor = managed conservatively with full graft retention.

## Data Availability

The datasets generated during and/or analyzed during the current research are available from the corresponding author upon reasonable request.

## Supplementary Materials

The following supporting information can be downloaded at: https://www.mdpi.com/article/10.3390/jcm14248639/s1, PRISMA 2020 Checklist.

## Abbreviations

The following abbreviations are used in this manuscript:

GBR	Guided Bone Regeneration
ST	Shell Technique
VRA	Vertical ridge augmentation
VBG	Vertical bone gain
PROMs	Patient-Reported Outcome Measures
Ti-PTFE	Titanium-reinforced polytetrafluoroethylene
Ti-mesh	Titanium mesh
d-PTFE	Dense polytetrafluoroethylene
CBCT	Cone beam computed tomography
AUG	Autogenous bone graft
ALG	Allogeneic bone graft
BS	Bone substitute
ICG	Iliac crest graft
IBB	Intraoral autogenous bone block
ABBs	Allogeneic bone blocks
CAD/CAM	Computer-aided design/computer-aided manufacturing
SBB	Split bone block
MP-ePTFE	Microporous expanded polytetrafluoroethylene
PES	Pink esthetic score
CTG	Connective tissue graft

## References

[1] Rocchietta I., Fontana F., Simion M. (2008). Clinical outcomes of vertical bone augmentation to enable dental implant placement: A systematic review. J. Clin. Periodontol..

[2] Urban I.A. (2023). Techniques on vertical ridge augmentation: Indications and effectiveness. Periodontology 2000.

[3] Terheyden H., Meijer G.J., Raghoebar G.M. (2021). Vertical bone augmentation and regular implants versus short implants in the vertically deficient posterior mandible: A systematic review and meta-analysis of randomized studies. Int. J. Oral Maxillofac. Surg..

[4] Maló P., de Araújo Nobre M., Lopes A., Ferro A., Moss S.M. (2019). The All-on-4 concept for full-arch rehabilitation of the edentulous maxillae: A longitudinal study with 5–13 years of follow-up. Clin. Implant Dent. Relat. Res..

[5] Lee C.T., Huang Y.W., Zhu L., Weltman R. (2016). Survival analysis of wide dental implants: A systematic review and meta-analysis. Clin. Oral Implants Res..

[6] Polido W.D., Machado-Fernandez A., Lin W.-S., Aghaloo T. (2023). Indications for zygomatic implants: A systematic review. Int. J. Implant Dent..

[7] Herce-López J., Pingarrón M.d.C., Tofé-Povedano Á., García-Arana L., Espino-Segura-Illa M., Sieira-Gil R., Rodado-Alonso C., Sánchez-Torres A., Figueiredo R. (2024). Customized subperiosteal implants for the rehabilitation of atrophic jaws: A consensus report and literature review. Biomimetics.

[8] Anitua E., Eguia A., Staudigl C., Alkhraisat M.H. (2024). Clinical performance of additively manufactured subperiosteal implants: A systematic review. Int. J. Implant Dent..

[9] Aceves-Argemí R., España-Tatay M., España-Tatay F., España-Tatay P. (2021). Titanium Meshes in Guided Bone Regeneration: A Systematic Review. Coatings.

[10] Nielsen H.B., Starch-Jensen T. (2021). Lateral Ridge Augmentation in the Posterior Part of the Mandible with an Autogenous Bone Block Graft Harvested from the Ascending Mandibular Ramus: A 10-Year Retrospective Study. J. Stomatol. Oral Maxillofac. Surg..

[11] Toledano-Serrabona J., Sánchez-Garcés M.Á., Sánchez-Torres A., Gay-Escoda C. (2019). Alveolar distraction osteogenesis for dental implant treatments of the vertical bone atrophy: A systematic review. Med. Oral Patol. Oral Cir. Bucal.

[12] Urban I.A., Montero E., Monje A., Sanz-Sánchez I. (2019). Effectiveness of vertical ridge augmentation interventions: A systematic review and meta-analysis. J. Clin. Periodontol..

[13] Zhou L., Su Y., Wang J., Wang X., Liu Q., Wang J. (2022). Effect of exposure rates with customized versus conventional titanium mesh on guided bone regeneration: Systematic review and meta-analysis. J. Oral Implantol..

[14] Garcia J., Dodge A., Luepke P., Wang H.L., Kapila Y., Lin G.H. (2018). Effect of membrane exposure on guided bone regeneration: A systematic review and meta-analysis. Clin. Oral Implants Res..

[15] Schwarz F., Ramanauskaite A., Wetzel W., Mayer S., Obreja K., Parvini P. (2024). Clinical Outcomes Following a Combined Vertical and Horizontal Bone Augmentation Procedure in the Posterior Mandible. Clin. Implant Dent. Relat. Res..

[16] Tunkel J., Hoffmann F., Schmelcher Y., Kloss-Brandstätter A., Kämmerer P.W. (2023). Allogeneic versus autogenous shell technique augmentation procedures: A prospective-observational clinical trial comparing surgical time and complication rates. Int. J. Implant Dent..

[17] Sáez-Alcaide L.M., González-Gallego B., Fernando-Moreno J., Navarro M.M., Cobo-Vázquez C., Brinkmann J.C.B., Meniz-García C. (2023). Complications associated with vertical bone augmentation techniques in implant dentistry: A systematic review of clinical studies published in the last ten years. J. Stomatol. Oral Maxillofac. Surg..

[18] Gotfredsen K. (2023). Patient-reported outcomes for bone regenerative procedures. Periodontology 2000.

[19] Kofina V., Monfaredzadeh M., Rawal S.Y., Dentino A.R., Singh M., Tatakis D.N. (2023). Patient-reported outcomes following guided bone regeneration: Correlation with clinical parameters. J. Dent..

[20] Page M.J., McKenzie J.E., Bossuyt P.M., Boutron I., Hoffmann T.C., Mulrow C.D., Shamseer L., Tetzlaff J.M., Akl E.A., Brennan S.E. (2021). The PRISMA 2020 Statement: An Updated Guideline for Reporting Systematic Reviews. BMJ.

[21] Tunkel J., Würdinger R., de Stavola L. (2018). Vertical 3D Bone Reconstruction with Simultaneous Implantation: A Case Series Report. Int. J. Periodontics Restor. Dent..

[22] Khoury F., Hanser T. (2019). Three-Dimensional Vertical Alveolar Ridge Augmentation in the Posterior Maxilla: A 10-Year Clinical Study. Int. J. Oral Maxillofac. Implants.

[23] Yu H., Chen L., Zhu Y., Qiu L. (2016). Bilaminar Cortical Tenting Grafting Technique for Three-Dimensional Reconstruction of Severely Atrophic Alveolar Ridge: A Prospective Study. J. Craniomaxillofac. Surg..

[24] Cucchi A., Vignudelli E., Napolitano A., Marchetti C., Corinaldesi G. (2017). Evaluation of Complication Rates and Vertical Bone Gain after Guided Bone Regeneration with Non-Resorbable Membranes versus Titanium Meshes and Resorbable Membranes. Clin. Implant Dent. Relat. Res..

[25] Khoury F., Hanser T. (2022). Three-Dimensional Vertical Alveolar Crest Augmentation: 10-Year Clinical Outcome. Int. J. Oral Implantol..

[26] Pabst A., Alshihri A., Becker P., Wurdinger R., Tunkel J., Kämmerer P.W. (2025). Vertical Bone Gain with the Allogeneic Shell Technique: Comparative Analysis of Surgical Approaches. J. Clin. Med..

[27] Tatli U., Cavana A., Tükel H.C., Benlidayi M.E. (2025). Effects of Bone Augmentation on Implant Success and Survival: A Retrospective Analysis with 6-Year Mean Follow-Up. Clin. Implant Dent. Relat. Res..

[28] Kämmerer P.W., Tunkel J., Götz W., Würdinger R., Kloss F., Pabst A. (2022). The Allogeneic Shell Technique for Alveolar Ridge Augmentation: A Multicenter Case Series. Int. J. Implant Dent..

[29] Mounir M., El Morsy O.A., Amer H., Mounir S., Gibaly A. (2021). Assessment of Bone Quality Using Buccal and Palatal Autogenous Cortical Shells Harvested from Two Donor Sites: A Randomized Clinical Trial. Oral Maxillofac. Surg..

[30] Scavia S., Roncucci R., Bianco E., Mirabelli L., Bader A., Madonna F. (2021). Vertical Bone Augmentation with GBR Pocket Technique: Surgical Procedure and Preliminary Results. J. Contemp. Dent. Pract..

[31] Hur Y., Ogata Y., Kim D.W., Pham C.M., Yoon T.H., Ogata H. (2017). Bone Resorption during Submerged Healing after Guided Bone Regeneration: A Prospective Case Series. Implant Dent..

[32] Mertens C., Büsch C., Goldenbaum K., Hoffmann J., Steveling H.G. (2023). Full Block or Split Block? Comparison of Two Different Autogenous Block Grafting Techniques for Alveolar Ridge Reconstruction. Clin. Implant Dent. Relat. Res..

[33] Gultekin B.A., Cansiz E., Borahan M.O. (2017). Clinical and Three-Dimensional Radiographic Evaluation of Autogenous Iliac Block Bone Grafting and Guided Bone Regeneration in Patients with Atrophic Maxilla. J. Oral Maxillofac. Surg..

[34] Cucchi A., Bettini S., Ghensi P., Fiorino A., Corinaldesi G. (2023). Vertical Ridge Augmentation with Ti-Reinforced Dense PTFE Membranes or Ti Meshes and Collagen Membranes: A Randomized Clinical Trial. Clin. Implant Dent. Relat. Res..

[35] Ji J.G., Yu J.A., Choi S.H., Lee D.W. (2021). Clinical, Radiographic, and Histomorphometric Evaluation of a Vertical Ridge Augmentation Procedure: Consecutive Case Series with 1-Year Follow-Up. Materials.

[36] Giragosyan K., Chenchev I., Ivanova V. (2024). Linear Bone Gain and Healing Complication Rate Following Ridge Augmentation with Titanium-Reinforced d-PTFE Membranes. Folia Med..

[37] Lee J.S., Park J.Y., Chung H.M., Song Y.W., Strauss F.J. (2022). Vertical Ridge Augmentation Feasibility Using Unfixed Collagen Membranes and Particulate Bone Substitutes: A Prospective Study. Clin. Implant Dent. Relat. Res..

[38] Shaker A.E.S., Salem A.S., El-Farag S.A.A., Abdel-Rahman F.H., El-Keiy M.M. (2024). Comparison of Khoury’s Bone Shell Technique vs Titanium-Reinforced d-PTFE Membranes: Randomized Clinical Trial. J. Contemp. Dent. Pract..

[39] Cucchi A., Sartori M., Parrilli A., Aldini N.N., Vignudelli E., Corinaldesi G. (2019). Histological and Histomorphometric Analysis of Bone Tissue after Guided Bone Regeneration with Non-Resorbable Membranes. Clin. Implant Dent. Relat. Res..

[40] Rokn A.R., Monzavi A., Panjnoush M., Hashemi H.M., Kharazifard M.J. (2018). Vertical Ridge Augmentation Using Resorbable Collagen Membranes: A Split-Mouth Clinical Trial. Clin. Implant Dent. Relat. Res..

[41] Restoy-Lozano A., Domínguez-Mompell J.L., Infante-Cossio P., Lara-Chao J., Espín-Gálvez F., López-Pizarro V. (2015). Reconstruction of Mandibular Vertical Defects for Dental Implants with Autogenous Bone Block Grafts Using a Tunnel Approach: Clinical Study of 50 Cases. Int. J. Oral Maxillofac. Surg..

[42] Cucchi A., Vignudelli E., Franco R., Marchetti C., Corinaldesi G. (2020). One-Year Peri-Implant Outcomes after Vertical Ridge Augmentation with Ti-Reinforced d-PTFE versus Ti Mesh with Collagen Membrane. Clin. Oral Implants Res..

[43] Esposito M., Grusovin M.G., Felice P., Karatzopoulos G., Worthington H.V., Coulthard P. (2009). Interventions for Replacing Missing Teeth: Horizontal and Vertical Bone Augmentation Techniques for Dental Implant Treatment. Cochrane Evid..

[44] Fontana F., Maschera E., Rocchietta I., Simion M. (2011). Clinical Classification of Complications in Guided Bone Regeneration Procedures by Means of a Nonresorbable Membrane. Int. J. Periodontics Restor. Dent..

[45] Sanz-Sánchez I., Sanz-Martín I., Ortiz-Vigón A., Molina A., Sanz M. (2022). Complications in bone-grafting procedures: Classification and management. Periodontology 2000.

[46] Barone A., Toti P., Menchini-Fabris G.B., Felice P., Marchionni S., Covani U. (2017). Early volumetric changes after vertical augmentation of the atrophic posterior mandible with interpositional block graft versus onlay bone graft: A retrospective radiological study. J. Craniomaxillofac. Surg..

[47] Pieroni S., Miceli B., Giboli L., Romano L., Azzi L., Farronato D. (2024). Efficacy of the Sausage Technique in Rebuilding the Crestal Buccal Bone Thickness: A Retrospective Analysis. Dent. J..

[48] Thoma D.S., Naenni N., Figuero E., Hämmerle C.H.F., Schwarz F., Jung R.E., Sanz-Sánchez I. (2018). Effects of Soft Tissue Augmentation Procedures on Peri-Implant Health or Disease: A Systematic Review and Meta-Analysis. Clin. Oral Implants Res..

[49] Chrcanovic B.R., Kisch J., Albrektsson T., Wennerberg A. (2016). Factors Influencing Early Dental Implant Failures. J. Dent. Res..

[50] Chrcanovic B.R., Albrektsson T., Wennerberg A. (2017). Bone Quality and Quantity and Dental Implant Failure. Implant Dent..

[51] Knöfler W., Barth T., Graul R., Krampe D. (2016). Retrospective analysis of 10,000 implants from insertion up to 20 years—Analysis of implantations using augmentative procedures. Int. J. Implant Dent..

[52] Papaspyridakos P., De Souza A., Vazouras K., Gholami H., Pagni S., Weber H.P. (2018). Survival rates of short dental implants (≤6 mm) compared with implants longer than 6 mm in posterior jaw areas: A meta-analysis. Clin. Oral Implants Res..

[53] Maiorana C., Poli P.P., Deflorian M., Testori T., Mandelli F., Nagursky H., Vinci R. (2017). Alveolar socket preservation with demineralised bovine bone mineral and a collagen matrix. J. Periodontal Implant Sci..

[54] Yu S.H., Saleh M.H.A., Wang H.L. (2023). Simultaneous or staged lateral ridge augmentation: A clinical guideline on the decision-making process. Periodontology 2000.

